# Effects of Arthroscopic Coracohumeral Ligament Release on Range of Motion for Patients with Frozen Shoulder

**DOI:** 10.2174/1874325001812010373

**Published:** 2018-09-18

**Authors:** Yoshihiro Hagiwara, Takuya Sekiguchi, Akira Ando, Kenji Kanazawa, Masashi Koide, Junichiro Hamada, Yutaka Yabe, Shinichiro Yoshida, Eiji Itoi

**Affiliations:** 1 Department of Orthopaedic Surgery, Tohoku University School of Medicine, 2-1 Seiryo-machi, Aoba-ku, Sendai, Japan.; 2 Department of Orthopaedic Surgery, Iwate Prefectural Centeral Hospital, Morioka, Japan; 3 Department of Otrhopaedic Surgery, Matsuda Hospital, Sendai, Japan.; 4 Department of Otrhopaedic Surgery, South Miyagi Medical Center, Ohgawara, Japan; 5 Department of Orthopaedic Surgery, Kuwano Kyoritsu Hospital, Koriyama, Japan

**Keywords:** Frozen shoulder, Adhesive capsulitis, Arthroscopic capsular release, Range of motion, Coracohumeral ligament, Joint capsule, Beach chair position, Goniometer

## Abstract

**Background:**

A thickened coracohumeral ligament is a highly specific manifestation of, and primary restraint against external rotation in frozen shoulders.

**Objective:**

The purpose of this study was to evaluate the effects of complete arthroscopic coracohumeral ligament release on range of motion in frozen shoulder.

**Methods:**

Fifty-two consecutive shoulders in 52 patients were treated between April 2015 and June 2016. To evaluate solely glenohumeral range of motion, the scapula was fixed by an examiner with one hand (without palpating scapular motion), and range of motion was measured using a goniometer. For the first step, arthroscopic pancapsular release was performed in a beach-chair position with (Group 1) or without (Group 2) complete coracohumeral ligament release. For the final step, the remaining coracohumeral ligaments in Group 2 were released and the ranges of motion were compared to those in Group 1.

**Results:**

The average age of the patients was significantly higher in Group 1, but there were no significant differences between the two groups with respect to sex, affected side, preoperative range of motion, or American Shoulder and Elbow Society Score. Abduction, external rotation at adduction, and external and internal rotations at 90° of flexion in Group 1 were significantly greater than those in Group 2. After the additional release of the remaining coracohumeral ligaments in Group 2, all ranges of motion were significantly recovered and there was no significant difference between the groups.

**Conclusion:**

Complete coracohumeral ligament release is a recommended intraoperative procedure for regaining full range of motion in frozen shoulders.

## 1 INTRODUCTION

Frozen shoulder is characterized by painful restriction of both active and passive Ranges of Motion (ROM) [1]. Even after a few years of conservative treatment, over 50% of patients with frozen shoulder have some persistent symptoms [2, 3]. In cases of limited ROM after appropriate conservative treatments such as oral medications, injections, and hydrodilation, or surgical interventions such as manipulation and capsular release [4, 5], additional treatment options should be considered.

Manipulation Under Anesthesia (MUA) can reduce the duration of symptoms and appears to be safer and more reliable than previously reported when adequate attention is paid to the potential complications [4, 6]. However, some studies have reported minimal changes after MUA, and have recommended arthroscopic capsular release for the recalcitrant frozen shoulder [7, 8]. Since, MUA cannot release the entire capsule including the Coracohumeral Ligament (CHL) [6, 9], the remaining thickened capsule may continue to restrict the ROM in some cases. This is especially true with movements such as internal rotation, which includes Hand Behind the Back (HBB) or Horizontal Flexion (HF) motions [10].

CHL originates from the base and horizontal limb of the coracoid process, enclosing the subscapularis, supraspinatus, and infraspinatus tendons [11]. A thickened CHL at the rotator interval is considered to be the most specific manifestation of frozen shoulder [12]. It is also the primary restraint against External Rotation (ER) in frozen shoulders [13-15]. Arthroscopic capsular release after consecutive MUA reveals the intact CHL from the base of the coracoid process to the superior capsule. Limited internal rotation is recovered just after its release [9]. In cases of recurrent anterior shoulder instability, subcoracoid fat triangle obliteration and CHL thickness were positively correlated with ROM restriction (Forward Flexion [FF] [16], ER, and HBB [17]). A contracted CHL restricts the sliding motions of the supraspinatus around the base of the coracoid process and the subscapularis under the coracoid process [10]. It has been reported that after a complete arthroscopic CHL release for frozen shoulder, ER and Internal Rotation (IR) were both recovered [10].

The purpose of this study was to evaluate the effects of complete arthroscopic CHL release on ROMs evaluated during surgery.

## 2 MATERIALS AND METHODS

This is a prospective case-control study. This study protocol was approved by the institutional review boards of Tohoku University School of Medicine (the approval number 2015-1-483). All patients provided informed consent for the study and the procedures.

## 3 INCLUSION AND EXCLUSION CRITERIA

The diagnosis of frozen shoulder comprised: (1) greater than a one-month history of shoulder pain and stiffness; (2) limited passive shoulder ROM of 100° or less in FF, 20° or less in ER with the arm at the side, and to the level of the 5th lumbar vertebra or less during HBB (measured by asking the patients to place the thumb on the highest spinal vertebrae they could possibly reach); and (3) normal radiographic appearance of the shoulder [3, 18]. Patients were excluded based on radiographic evidence of any of the following: abnormalities indicating glenohumeral osteoarthritis, calcific tendinitis, superiorly migrated humeral head, osteonecrosis of the humeral head, or rotator cuff tears visualized on magnetic resonance imaging. Patients with a history of previous fractures around the shoulder, dislocation of the shoulder, thyroid disorders, diabetes mellitus, and post-traumatic frozen shoulder were also excluded.

### 3.1 Preoperative Treatment

Between April 2015 and June 2016, patients with frozen shoulder were treated using physiotherapy and steroid injections for pain relief. A mixture of 4 mg of dexamethasone and 10 ml of 1% lidocaine was injected under ultrasonographic guidance until symptom relief occurred (≤2 injections in total, at a frequency of 1 injection/week). Stretching of the muscles around the shoulder girdle, thorax, spine, trunk, and hip joints was performed [10]. If limited ROM still remained after at least three months of physiotherapy, an arthroscopic capsular release was recommended.

### 3.2 ROM Measurement

A total of 52 consecutive shoulders in 52 patients were included in this study (Table **1**) and were divided into two groups. To evaluate the true glenohumeral ROM and exclude scapular-thoracic motion, the scapula was first fixed by an examiner with one hand (without palpating scapular motion), and the following motions were measured in a beach-chair position under general anesthesia using a goniometer: passive ROM of FF, abduction (ABD), ER with the arm at side (1^st^ ER), ER at 90° of abduction (2^nd^ ER), IR at 90° of abduction (2^nd^ IR), and HF, ER at 90° of FF (3^rd^ ER), and IR at 90° of FF (3^rd^ IR) [10]. In cases of difficulty with 90° of FF or ABD, the ROMs were evaluated at the maximum degrees of FF and ABD. In Group 1, a conventional arthroscopic capsular release with an entire CHL release was performed and the final ROM was measured [10]. In Group 2, after a conventional arthroscopic capsular release without complete CHL release, the ROM was evaluated. Subsequently, the remaining CHL around the base and under the coracoid process was released arthroscopically, and the ROM was evaluated again at the final step [10]. All patients were placed in a beach-chair position and the ROMs were evaluated after removing the arthroscope. The surgical bed was set in horizontal fashion, and the angle between the patient’s trunk and the surgical bed was measured with a goniometer prior to draping. After draping, the axis of the trunk was recreated with a surgical pen before starting the operation. There were no complications during or after the operations and no cases of rotator cuff abnormalities. All surgical procedures and ROM measurements were performed by a single surgeon (Y.H.), and were performed without subacromial decompression.

### 3.3 Statistical Analysis

Continuous variables are presented as means and standard derivations, and categorical variables (sex and affected side) are presented as numbers and percentages (%). The Student’s t test was used for comparisons of age and ROMs (FF, ABD, 1^st^ ER, 2^nd^ ER, 2^nd^ IR, HF, 3^rd^ ER, and 3^rd^ IR). The Mann-Whitney U test was conducted to compare the American Shoulder and Elbow Society Shoulder scores (ASES score) and the Chi-squared test was conducted to compare categorical variables. An analysis of covariance based on age and sex was used to compare the ROMs under general anesthesia between the two groups. All p-values <0.05 were considered statistically significant. Statistical analyses were performed using the statistical software package SPSS for Windows (version 23.0, SPSS Inc., Chicago, IL, USA).

## 4 RESULTS

There were no significant differences between the two groups with regard to sex, affected side, or ASES score (pain and function). The mean age of Group 1 was greater than that of Group 2 (Table **1**). There were no significant differences in ROMs between the two groups just before surgery. The ROMs in Group 1 were greater than those in Group 2 in all directions at the first step, but the differences were significant in ABD, 1^st^ ER, 3rd ER and IR. After the additional release of the remaining CHL in Group 2, ROMs in all directions were significantly recovered and there were no significant differences between the two groups after the final step (Table **2**).

## 5 DISCUSSION

The most important finding in this study was that a contracted CHL restricted ROM in ABD, 1^st^ ER, 3^rd^ ER, and 3^rd^ IR. Complete CHL release is recommended for regaining full ROM of the frozen shoulder.

A thickened CHL is one of the most specific manifestations of [9], and primary restraint against ER for the frozen shoulder [13-15]. In this study, we found that CHL restricted the ROMs of ABD, 1^st^ ER, 3^rd^ ER, and 3^rd^ IR. The restriction of the 1^st^ ER in this study was consistent with previous reports, and release of the CHL under the coracoid process allowed smooth movement of the subscapularis tendon [10]. However, there are no previous reports on the restriction of ROMs in ABD, 3^rd^ ER, and 3^rd^ IR due to a thickened CHL. These three movement directions could represent specific ROM restrictions with thickened CHLs.

Restriction of IR of the shoulder joint is believed to be related to posterior capsular tightness in athletes that perform throwing motions [19, 20]. However, IR with the arm at the side is retained in most patients with a frozen shoulder, which indicates that the thickened posterior capsule is not the only factor causing the restriction of IR. Furthermore, MUA with a controlled force to the humerus induced a rupture of the anteroinferior portion of the glenohumeral ligament. The findings of another study indicated that after arthroscopic release of a thickened CHL with an intact superomedial capsule, the 3^rd^ IR recovered to a level that was similar to the unaffected side [9], which corresponds well with the findings of this study. The CHL and the superomedial capsule play an important role in ROM limitations, especially in the 3^rd^ IR.

The CHL originates from the base and horizontal limb of the coracoid process, covering the subscapularis, supraspinatus, and infraspinatus tendons [11]. Based on recent study findings, the CHL covers a greater area than previously reported [21]. The CHL is divided into two parts according to macroscopic evaluation. One part of the CHL spreads its fibers over the rotator interval to the posterior portion of the greater tuberosity of the humerus. The other part envelops the superior portion of the subscapularis muscle. The anterior CHL holds the subscapularis muscle and anchors the muscle to the coracoid process in a similar manner to the posterior CHL, which envelops the supraspinatus [21]. Contractures or adhesions in these areas could lead to an inhibition of the sliding mechanism of the supraspinatus and subscapularis tendons, thus altering the rotational center between the glenoid cavity and the humeral head. Further biomechanical studies are needed to clarify this motion effect.

Conventional ROM of the shoulder comprises a combined motion of the glenohumeral joint and the scapulothoracic joint. However, measuring the true ROM of the glenohumeral joint is necessary to evaluate the capsular effects on ROM [10]. Because the scapula is semi-fixed in a beach-chair position, it is difficult to precisely evaluate conventional ROM. Evaluation of the true ROM of the glenohumeral joint with scapular fixation is a reliable evaluation method during surgery, as well as in the outpatient clinic [10].

This study has several limitations. First, the surgeries and ROM evaluations were performed by a single surgeon, and the degree of tissue expansion during surgery was not estimated. Future research should include the performance of reliability tests, such as inter-rater reliability testing. Second, the pre- and postoperative evaluations of ROM performed in the outpatient clinic were not included and randomization was not adopted. Third, the long-term clinical outcomes of the entire arthroscopic capsular release were not evaluated.

## CONCLUSION

A complete arthroscopic CHL release around the base, as well as under the coracoid process, is a recommended intraoperative procedure for regaining full ROM in a frozen shoulder, especially the ROMs in ABD, 1^st^ ER, 3^rd^ ER, and 3^rd^ IR.

## Figures and Tables

**Table 1:** Baseline characteristics.

	Group 1	Group 2	*P-Value*
Number of patients	24	28	
Age	61.6 (6.6)	56.3 (7.8)	0.012
Sex (woman)	12 (50.0)	4 (50.0)	1
Affected side (left)	15 (62.5)	20 (71.4)	0.56
ASES score (total)	40.4 (16.0)	42.6 (15.2)	0.62
pain	20.0 (7.2)	24.1 (8.7)	0.073
function	19.6 (9.9)	18.5 (10.1)	0.69

ASES scores: American Shoulder and Elbow Society Shoulder scores.

**Table 2:** Range of motion with or without coracohumeral release.

		Group 1 (n = 24)	Group 2 (n = 28)	*P-Value*	Partial η2	F (1, 47)
Pre-operation	FF	72.5 (13.4)	69.6 (12.1)	0.75	0.002	0.11
	ABD	64.6 (19.8)	67.3 (17.1)	0.29	0.024	1.14
	1st ER	-9.8 (19.0)	-8.6 (20.0)	0.7	0.003	0.15
	2nd ER	59.2 (13.0)	55.5 (16.2)	0.56	0.007	0.34
	2nd IR	-44.2 (8.7)	-43.4 (13.3)	0.55	0.008	0.37
	HF	62.9 (15.0)	58.6 (15.7)	0.8	0.001	0.065
	3rd ER	72.3 (12.9)	65.9 (14.4)	0.18	0.039	1.89
	3rd IR	-55.6 (11.8)	-51.6 (11.9)	0.11	0.053	2.64
Pancapsular release	FF	151.0 (21.6)	138.9 (24.1)	0.069	0.069	3.46
	ABD	162.1 (17.6)	149.5 (26.2)	0.044	0.084	4.29
	1st ER	68.8 (18.3)	58.0 (18.7)	0.031	0.096	4.97
	2nd ER	115.6 (14.1)	110.5 (20.3)	0.29	0.023	1.13
	2nd IR	9.6 (10.5)	3.6 (15.0)	0.11	0.053	2.65
	HF	134.6 (13.1)	133.6 (18.9)	0.87	0.001	0.028
	3rd ER	102.1 (11.4)	92.0 (8.4)	<0.001	0.26	16.4
	3rd IR	1.9 (5.3)	-32.9 (12.7)	<0.001	0.75	138.01
Additonal CHL release	FF		152.0 (24.5)	0.91*	<0.001	0.012
	ABD		159.6 (26.6)	0.57*	0.007	0.34
	1st ER		70.7 (18.1)	0.85*	0.001	0.037
	2nd ER		118.4 (18.9)	0.85*	0.003	0.13
	2nd IR		15.2 (10.0)	0.85*	0.057	2.82
	HF		138.9 (18.2)	0.85*	0.013	0.64
	3rd ER		97.7 (8.8)	0.85*	0.071	3.58
	3rd IR		1.6 (5.9)	0.85*	0.007	0.33

Abbreviations: FF: forward flexion; ABD: abduction; 1st ER: external rotation with the arm at side; 2^nd^ ER: external rotation at 90° of abduction; 2^nd^ IR: internal rotation at 90°of abduction; HF: horizontal flexion; 3^rd^ ER: external rotation at 90°of forward flexion; 3^rd^ IR: internal rotation at 90°of forward flexion * compared with Group 1

## LIST OF ABBREVIATIONS

1^st^ ER: ER with the arm at side
2^nd^ ER: ER at 90º of abduction
2^nd^ IR: IR at 90º of abduction
3^rd^ ER: ER at 90º of forward flexion
3^rd^ IR: IR at 90º of forward flexion
ABD: Abduction
CHL: Coracohumeral Ligament
ER: External Rotation
FF: Forward Flexion
HBB: Hand Behind the Back
HF: Horizontal Flexion
IR: Internal Rotation
MUA: Manipulation Under Anesthesia
ROM: Range of Motion
